# Chronotropic incompetence and myocardial injury after noncardiac surgery: planned secondary analysis of a prospective observational international cohort study

**DOI:** 10.1016/j.bja.2019.03.022

**Published:** 2019-04-24

**Authors:** Tom E.F. Abbott, Rupert M. Pearse, W. Scott Beattie, Mandeep Phull, Christian Beilstein, Ashok Raj, Michael P.W. Grocott, Brian H. Cuthbertson, Duminda Wijeysundera, Gareth L. Ackland

**Affiliations:** 1 William Harvey Research Institute, Queen Mary University of London, London, UK; 2 Department of Anesthesia, University of Toronto, Toronto, ON, Canada; 3 Department of Intensive Care Medicine, Queens Hospital, Romford, UK; 4 Department of Anaesthesiology and Pain Therapy, Bern University Hospital, Bern, Switzerland; 5 Department of Intensive Care Medicine, Croydon University Hospital, Croydon, UK; 6 Critical Care Research Group, NIHR Southampton Biomedical Research Centre, University Hospital Southampton, University of Southampton, Southampton, UK; 7 Department of Critical Care Medicine, Sunnybrook Health Sciences Centre, Toronto, ON, Canada; 8 Li Ka Shing Knowledge Institute, St Michael's Hospital, Toronto, ON, Canada

**Keywords:** autonomic dysfunction, general surgery, myocardium, sympathetic, vagus

## Abstract

**Background:**

Physiological measures of heart failure are common in surgical patients, despite the absence of a diagnosis. Heart rate (HR) increases during exercise are frequently blunted in heart failure (termed *chronotropic incompetence*), which primarily reflects beta-adrenoreceptor dysfunction. We examined whether chronotropic incompetence was associated with myocardial injury after noncardiac surgery.

**Methods:**

This was a predefined analysis of an international cohort study where participants aged ≥40 yr underwent symptom-limited cardiopulmonary exercise testing before noncardiac surgery. Chronotropic incompetence was defined as the ratio of increase in HR during exercise to age-predicted maximal increase in HR <0.6. The primary outcome was myocardial injury within 3 days after surgery, defined by high-sensitivity troponin assays >99th centile. Explanatory variables were biomarkers for heart failure (ventilatory efficiency slope [minute ventilation/carbon dioxide production] ≥34; peak oxygen consumption ≤14 ml kg^−1^ min^−1^; HR recovery ≤6 beats min^−1^ decrease 1 min post-exercise; preoperative N-terminal pro-B-type natriuretic peptide [NT pro-BNP] >300 pg ml^−1^). Myocardial injury was compared in the presence or absence of sympathetic (i.e. chronotropic incompetence) or parasympathetic (i.e. impaired HR recovery after exercise) thresholds indicative of dysfunction. Data are presented as odds ratios (ORs) (95% confidence intervals).

**Results:**

Chronotropic incompetence occurred in 396/1325 (29.9%) participants; only 16/1325 (1.2%) had a heart failure diagnosis. Myocardial injury was sustained by 162/1325 (12.2%) patients. Raised preoperative NT pro-BNP was more common when chronotropic incompetence was <0.6 (OR: 1.57 [1.11–2.23]; *P*=0.011). Chronotropic incompetence was not significantly associated with myocardial injury (OR: 1.05 [0.74–1.50]; *P*=0.78), independent of rate-limiting therapy. HR recovery <12 beats min^−1^ decrease after exercise was associated with myocardial injury in the presence (OR: 1.62 [1.05–2.51]; *P*=0.03) or absence (OR: 1.60 [1.06–2.39]; *P*=0.02) of chronotropic incompetence.

**Conclusions:**

Chronotropic incompetence is common in surgical patients. In contrast to parasympathetic dysfunction which was associated with myocardial injury, preoperative chronotropic incompetence (suggestive of sympathetic dysfunction) was not associated with postoperative myocardial injury.

**Editor's key points**

• Many moderate- to high-risk surgical patients have cardiac autonomic dysfunction, which can manifest as either parasympathetic or sympathetic dysfunction, or as a combined disorder.
• The inability to increase HR sufficiently quickly during exercise (chronotropic incompetence) reflects sympathetic dysfunction, and failure to decrease HR sufficiently quickly after termination of exercise (impaired HR recovery) points to parasympathetic dysfunction.
• In this multicentre cohort study, including 1325 moderate- to high-risk noncardiac surgery patients, all of whom underwent cardiopulmonary exercise testing, approximately 30% had preoperative chronotropic incompetence and 40% had impaired HR recovery.
• Chronotropic incompetence (sympathetic dysfunction) was related to preoperative biomarker indicators of heart failure, but, when adjusting for known confounders, was not significantly associated with 3-day postoperative myocardial injury or 1-yr mortality.
• In contrast, in a multivariable analysis, preoperative impaired HR recovery (parasympathetic dysfunction), in isolation or in combination with chronotropic incompetence, was significantly associated with 3-day postoperative myocardial injury.

Around 30% of patients undergoing noncardiac surgery sustain asymptomatic myocardial injury, which is strongly associated with mortality during hospital admission.^1^, ^2^, ^3^ Myocardial injury is more likely to occur in patients with preoperative cardiac vagal (parasympathetic) dysfunction, identified by impaired HR recovery (i.e. decrease) after exercise.^4^ Cardiac vagal autonomic impairment is a common feature in deconditioned surgical patients,^5^, ^6^, ^7^ in whom preoperative cardiopulmonary exercise testing (CPET) also reveals physiological features of cardiac failure, including lower peak oxygen consumption and higher resting HR.^8^

Whilst cardiac vagal activity reduces HR after exercise, maximal aerobic exercise is facilitated by increases in HR, principally driven by the sympathetic nervous system.^9^, ^10^, ^11^, ^12^, ^13^, ^14^ The impaired ability to increase HR,^4^ which is required for increased activity or demand, is broadly defined as chronotropic incompetence.^12^ In cardiac failure, high circulating levels of catecholamines result in decreased β-adrenoceptor density and desensitisation, which limit β-agonist-mediated contractility.^15^ Consequently, chronotropic incompetence is a robust predictor of mortality in patients with overt, clinically diagnosed cardiac failure.^12^

Here, we hypothesised that chronotropic incompetence, identified during preoperative CPET, was associated with myocardial injury within 3 days after noncardiac surgery. In order to assess whether sympathetic or parasympathetic dysfunction was more strongly associated with myocardial injury, we also evaluated the relationships between impaired (i) HR increase with exercise (i.e. chronotropic incompetence), and (ii) HR recovery from exercise and myocardial injury.

## Methods

### Study design and setting

This was a predefined secondary analysis of the Measurement of Exercise Tolerance before Surgery (METS) study, an international, prospective, observational cohort study of preoperative assessment before noncardiac surgery at 25 hospitals in the UK, Canada, New Zealand, and Australia. The study protocol and the main study results were published previously.^2^, ^16^ Research ethics committees reviewed the study, and it was conducted in accordance with the principles of the Declaration of Helsinki and the Research Governance Framework.

### Participants

Participants were aged 40 yr or older, undergoing elective noncardiac surgery under general anaesthesia or regional anaesthesia with a planned overnight stay in a hospital, and with at least one of the following perioperative risk factors: intermediate- or high-risk surgery, coronary artery disease, heart failure, cerebrovascular disease, diabetes mellitus, preoperative renal insufficiency, peripheral arterial disease, hypertension, a history of tobacco smoking within the previous year, or be aged 70 yr or older. The exclusion criteria were planned procedure using only endovascular technique, use of CPET for risk stratification as part of routine care, insufficient time for CPET before surgery, presence of an implantable cardioverter defibrillator, known or suspected pregnancy, previous enrolment in the study, severe hypertension (systolic pressure >180 mm Hg or diastolic pressure >100 mm Hg), active cardiac conditions, or other contraindications precluding CPET.^16^, ^17^ The participants gave written informed consent to take part before surgery.

### Study conduct and data collection

Researchers collected data directly from participants and their medical record. A detailed and standardised data set was collected before surgery, during the hospital stay, and after surgery. One year after surgery, the participants were contacted by telephone and underwent a short interview. Each participant underwent CPET and had blood sampled for N-terminal pro-B-type natriuretic peptide (NT pro-BNP) before surgery, and routine blood sampling for cardiac troponin on the 1st, 2nd, and 3rd days after surgery.

### Cardiopulmonary exercise testing

The participants underwent preoperative symptom-limited CPET using a standardised incremental ramp protocol with electromagnetically braked cycle ergometers.^18^ The test protocol consisted of spirometry in the seated position, followed by 3 min of rest sitting on the ergometer, followed by 3 min of unloaded pedalling, followed by pedalling with progressively increasing workload. Once the participants reached their peak performance, the exercise test was stopped, the workload reduced to 20 W, and the participants continued to pedal for 5 min in order to cool down. The participants were encouraged to pedal at a steady rate of 60 rev min^−1^. Work rates increased by 10 W min^−1^ in untrained participants, and by 20–30 W min^−1^ in trained participants or those undertaking regular physical activity according to a specific algorithm. Cardiopulmonary function was monitored continuously via electrocardiogram; pulse oximetry; and breath-by-breath measurement of minute ventilation, carbon dioxide production, and oxygen consumption. Non-invasive blood pressure was monitored every 3 min. The investigators at each site interpreted each CPET and collected a standardised data set. Peak oxygen consumption was calculated as the mean oxygen consumption during the final 20 s of incremental exercise.^19^ The anaerobic threshold was identified using the modified V-slope method, followed by the ventilatory equivalent and excess carbon dioxide methods.^20^ Clinicians at each site were blinded to the results of CPET, except where there was a safety concern according to predefined criteria.^16^

### Exposures

The exposure of interest was chronotropic incompetence, defined as chronotropic index (CI) <0.6 using the method described by Dobre and colleagues.^21^ This threshold is associated with mortality in patients with severe heart failure.^21^ CI is the ratio of measured increase in HR during exercise to the age-predicted maximal increase in HR.^12^ HR was measured at rest and at peak oxygen consumption during CPET to give the measured increase in HR. The most widely accepted method for calculating age-predicted maximal HR is 220–age, as described by Astrand.^22^, ^24^ The CI for the main analysis was calculated using the formula:

(1) CI=(peak HR–resting HR)/(age-predicted maximal HR–resting HR)

However, as various population-dependent thresholds have been derived,^23^ it has been suggested to use a CI equation generated in a population most closely matching the population of interest. The equation suggested by Tanaka and colleagues^23^ is recommended for apparently healthy persons, whilst other equations are recommended for those with known or suspected cardiovascular disease. In this study, we primarily used the Astrand^24^ method, and supplemented this with two *post hoc* sensitivity analyses: firstly, the calculated CI using the Tanaka and colleagues^23^ method, and second, using the CI (Astrand^24^) as a continuous variable.

### Primary outcome

The primary outcome measure was myocardial injury, defined as blood troponin T or I concentration greater than the limit of the reference range (99th centile) for each assay, within 72 h after surgery. Troponin assays differed between participating hospitals, and are listed in Supplementary Table S1. The secondary outcome was all-cause mortality at 1 yr after surgery. Predefined explanatory variables were preoperative NT pro-BNP >300 pg ml^−1^, a threshold used to predict postoperative cardiovascular events in surgical patients^25^ and heart failure in community cohorts,^26^ and preoperative CPET-derived markers of subclinical heart failure (ventilatory efficiency slope [minute ventilation/carbon dioxide production] ≥34, peak oxygen consumption ≤14 ml kg^−1^ min^−1^, and HR recovery ≤6 beats min^−1^ decrease at 1 min after the end of exercise).^8^

### Statistical analysis

We used STATA version 14 (StataCorp LP, College Station, TX, USA) to analyse the data. We excluded the small number of participants without a record of the exposure or outcome. We ranked the sample by CI and dichotomised it according to a threshold of <0.6, to define groups with and without chronotropic incompetence. We presented the baseline characteristics for the whole cohort and stratified by chronotropic incompetence. Firstly, we used univariable logistic regression analysis to test for the association between chronotropic incompetence and myocardial injury. Secondly, we constructed multivariable logistic regression models, adjusted for co-variates that are known to be associated with perioperative myocardial injury and routinely used for preoperative risk assessment: age >70 yr, male sex, preoperative renal insufficiency, peripheral vascular disease, existing diagnosis of heart failure, coronary artery disease, hypertension, diabetes mellitus, chronic obstructive pulmonary disease, cerebrovascular disease, high-risk surgery, and pre-existing atrial fibrillation.^27^, ^28^, ^29^, ^30^, ^31^, ^32^ We used backwards stepwise selection to identify variables for inclusion in the final model, with a Type 1 error threshold of <0.1. Missing data were handled by list-wise deletion. The results of logistic regression analyses were presented as odds ratios (ORs) with 95% confidence intervals. Normally distributed data were expressed as mean (standard deviation), and non-normally distributed data were expressed as median (inter-quartile range). Binary data were expressed as percentages. The threshold for statistical significance was *P*<0.05.

### Secondary analyses

We repeated the primary analysis using mortality within 1 yr after surgery, a binary categorical variable, as the outcome measure. We previously described a relationship between preoperative resting HR and subclinical heart failure.^8^ To explore whether chronotropic incompetence is associated with a phenotype of heart failure in this cohort, we repeated the primary analysis using the following outcome measures, which are biomarkers known to be predictive of poor clinical outcome in overt heart failure: NT pro-BNP >300 pmol L^−1^, peak oxygen consumption ≤14 ml kg^−1^ min^−1^, ventilatory efficiency slope (minute ventilation/carbon dioxide production) at the anaerobic threshold ≥34, and HR recovery ≤6 beats min^−1^ decrease.^8^

We have demonstrated previously that parasympathetic autonomic dysfunction is associated with myocardial injury.^4^ In order to draw direct comparisons between sympathetic and parasympathetic dysfunction, we examined the prevalence of physiological markers of impaired sympathetic and parasympathetic functions using chronotropic incompetence and HR recovery, respectively. We used a widely accepted definition of parasympathetic dysfunction, HR recovery <12 beats min^−1^ decrease during the first minute after the end of exercise.^9^

### Sensitivity analyses

Resting HR and the HR response to exercise can be influenced by medications, such as beta blockers and rate-limiting calcium channel antagonists, which may influence the results of our analysis. We addressed this in three ways. Firstly, we repeated the primary analysis, including beta blockers and diltiazem/verapamil as co-variates in the multivariable model. Secondly, we repeated the primary analysis, excluding patients receiving beta blockers and diltiazem/verapamil. Thirdly, we examined whether the use of beta blockade/calcium channel blocker altered the participants' ability to exceed a respiratory exchange ratio (RER) >1.05, as RER <1.05 indicates sub-maximal effort, or that the test was terminated prematurely.^12^

Our main analysis used the Astrand^24^ method. We also performed a *post hoc* sensitivity analysis, which repeated the primary analysis using age-predicted maximal HR calculated by the Tanaka and colleagues^23^ method, as we suspect that a substantial number of surgical patients have subclinical cardiac failure.^8^ We primarily defined chronotropic incompetence as CI <0.6, as described in studies of patients with heart failure. However, studies in other populations have defined chronotropic incompetence as CI <0.8.^33^ Therefore, we repeated the primary analysis using CI <0.8 as the exposure, and examined the CI as a continuous variable.

### Sample size estimation

As this was a planned secondary analysis of prospectively collected data, the sample size was determined based on the comparisons being made in the principal analysis, which has been published previously.^2^ We estimated that chronotropic incompetence may be present in up to ∼30% participants. Overall, 12.6% of participants in METS sustained perioperative myocardial injury. If participants with chronotropic incompetence had a higher incidence of ∼16%, at least 1305 participants' data would be required to detect a clinically significant difference (α=0.05; 1-β=80%).

## Results

**Fig 1:**
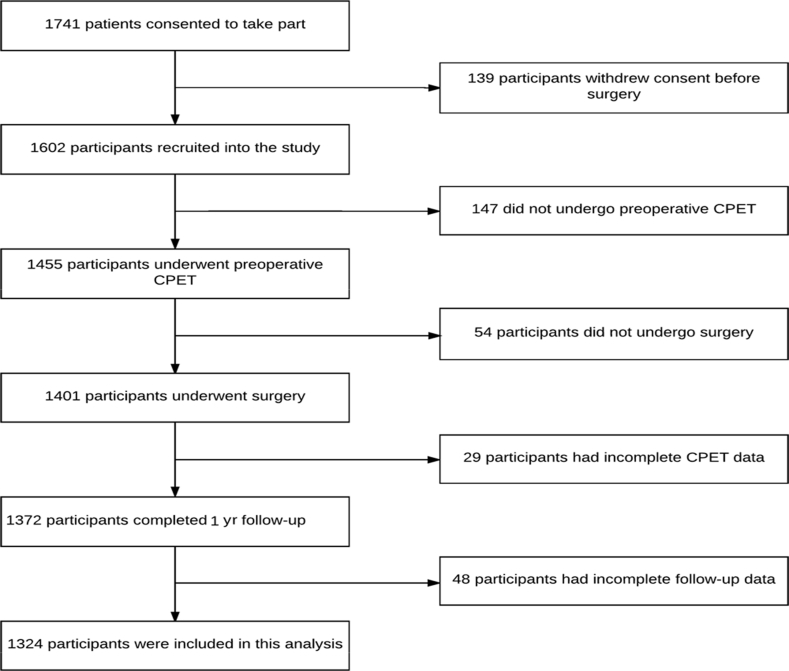
Patient flow diagram showing the number of cases included in the analysis. CPET, cardiopulmonary exercise testing.

Patients (1741) were recruited into the METS study between March 1, 2013 and March 25, 2016. After predefined exclusions of patients, we analysed data obtained from 1325 participants (Fig. 1). CI <0.6 was present in 396/1325 (29.9%) study participants, of whom 816/1325 (61.7%) were male (Table 1).

**Table 1:** Baseline patient characteristics. Descriptive data stratified by preoperative chronotropic incompetence (defined as chronotropic index [CI] <0.6). Data are presented as *n* (%) or mean (standard deviations; sd). Continuous data are reported to one decimal place, and categorical data are rounded to the nearest whole number.

	Whole cohort	CI <0.6	CI ≥0.6
Number of cases, *n*	1324	396	928
Mean age (sd)	64.2 (10.4)	64.8 (10.5)	64.0 (10.3)
Age ≥70 yr (%)	447 (33.8)	149 (37.6)	298 (32.1)
Male sex (%)	817 (61.7)	236 (59.6)	581 (62.6)
Co-morbid disorder (%)			
Atrial fibrillation	50 (3.8)	23 (5.8)	27 (2.9)
Diabetes mellitus	243 (18.4)	90 (22.7)	153 (16.5)
Hypertension	725 (54.8)	238 (60.1)	487 (52.5)
Diagnosis of congestive cardiac failure	16 (1.2)	10 (2.5)	6 (0.7)
Coronary artery disease	153 (11.6)	72 (18.2)	81 (8.7)
Peripheral vascular disease	39 (3.0)	18 (4.6)	21 (2.3)
Previous stroke or transient ischaemic attack	52 (3.9)	25 (6.3)	27 (2.9)
Chronic obstructive pulmonary disease	163 (12.3)	74 (18.7)	89 (9.6)
Preoperative estimated glomerular filtration rate <60 ml min^−1^ (1.73 m^2^)^−1^	108 (8.2)	45 (11.4)	63 (6.8)
Surgical procedure type (%)			
Vascular	23 (1.7)	12 (3.0)	11 (1.2)
Intraperitoneal or retroperitoneal	29 (2.2)	5 (1.3)	24 (2.6)
Urological or gynaecological	437 (33.0)	131 (33.1)	306 (33.0)
Intra-thoracic	306 (23.3)	107 (27.0)	199 (21.4)
Orthopaedic	398 (30.1)	106 (26.8)	292 (31.5)
Head and neck	87 (6.6)	23 (5.8)	64 (6.9)
Other	39 (3.0)	11 (2.8)	28 (3.0)
High-risk surgery (%)	756 (57.1)	221 (55.8)	535 (57.7)
ASA physical status (%)			
1	99 (7.5)	24 (6.1)	75 (8.1)
2	772 (58.4)	207 (52.3)	565 (61.0)
3	433 (32.8)	159 (40.2)	274 (29.6)
4	18 (1.4)	6 (1.5)	12 (1.3)
Preoperative medication (%)			
Beta blockers	213 (16.1)	137 (34.6)	76 (8.2)
Diltiazem or verapamil	25 (1.9)	11 (2.8)	14 (1.5)
Haemodynamic variables			
Resting heart rate (beats min^−1^)	77 (14.3)	75 (15.2)	78 (3.7)
Resting systolic blood pressure (mm Hg)	129 (18.1)	127 (9.0)	130 (17.6)
Resting pulse pressure (mm Hg)	51 (16.5)	51 (17.9)	52 (15.8)

### Markers of severe cardiac failure

Chronotropic index <0.6 was associated with elevated preoperative NT pro-BNP >300 pg ml^−1^ (OR: 1.57 [1.11–2.23]; *P*<0.001), adjusted for potentially confounding factors. CI <0.6 was also associated with three independent measures of moderate-to-severe heart failure (Table 3). CI <0.6 was more commonly found in patients with ventilatory efficiency slope (minute ventilation/carbon dioxide production) ≥34 (OR: 1.40 [1.09–1.81]; *P*=0.009), peak oxygen consumption ≤14 ml kg^−1^ min^−1^ (OR: 7.57 [5.50–10.43]; *P*<0.001), and HR recovery ≤6 beats min^−1^ decrease during the first minute after the end of exercise (OR: 2.63 [1.97–3.52]; *P*<0.001).

**Table 2:** Chronotropic incompetence and 1 yr mortality. The independent variable was chronotropic incompetence (defined as chronotropic index [CI] <0.6). The dependent variable was mortality within the 1 yr follow-up period. Results of two separate analyses are presented: firstly, univariable (unadjusted) logistic regression analysis, and secondly, multivariable logistic regression adjusting for three variables found to be associated with the dependent variable. The following variables were excluded from the final multivariable model: diabetes mellitus, peripheral vascular disease, atrial fibrillation, high-risk surgery, previous stroke or transient ischaemic attack, clinical diagnosis of heart failure, and preoperative renal insufficiency. Results are presented as odds ratios with 95% confidence intervals and associated *P*-values.

Co-variates	Mortality	
	Odds ratio	*P*-value
Univariable analysis		
Chronotropic incompetence	2.26 (1.13–4.51)	0.02
Multivariable analysis		
Male sex	2.22 (0.98–5.03)	0.06
History of stroke or transient ischaemic attack	3.00 (0.99–9.03)	0.05
History of chronic obstructive pulmonary disease	2.61 (1.17–5.86)	0.02
CI <0.6	1.98 (0.97–4.02)	0.06

**Table 3:** Chronotropic incompetence and markers of heart failure. The independent variable was chronotropic incompetence (defined as chronotropic index [CI] <0.6). The dependent variables were N-terminal pro-B-type natriuretic peptide (NT pro-BNP) >300 pg ml^−1^, ventilatory equivalent for carbon dioxide (VE/VCO_2_) at the anaerobic threshold ≥34, peak oxygen consumption (VO_2_) ≤14 ml kg^−1^ min^−1^, and HR recovery (HRR) ≤6 beats min^−1^ decrease within the first minute after the end of exercise. Results of univariable (unadjusted) and multivariable (adjusted) logistic regression analyses are presented as odds ratios with 95% confidence intervals and associated *P*-values. Variables were selected for inclusion in the multivariable model using stepwise selection.

Co-variates	NT pro-BNP >300 pmol L^−1^		VE/VCO_2_ ≥34		VO_2_ peak ≤14 ml kg^−1^ min^−1^		HRR ≤6 beats min^−1^	
	Odds ratio	*P*-value	Odds ratio	*P*-value	Odds ratio	*P*-value	Odds ratio	*P*-value
Univariable analysis								
CI <0.6	2.11 (1.56–2.86)	<0.001	1.57 (1.23–2.00)	<0.001	6.44 (4.82–8.59)	<0.001	2.81 (2.11–3.74)	<0.001
Multivariable analysis								
Age ≥70 yr	2.82 (2.01–3.95)	<0.001	2.58 (2.02–3.29)	<0.001	1.33 (0.95–1.84)	0.093	1.53 (1.13–2.05)	0.005
Male sex	—	—	0.55 (0.44–0.71)	<0.001	0.17 (0.12–0.23)	<0.001	0.63 (0.47–0.84)	0.002
History of atrial fibrillation	11.43 (5.71–22.88)	<0.001	—	—	—	—	—	—
History of heart failure	7.42 (2.01–27.40)	0.003	—	—	—	—	—	—
History of coronary artery disease	2.56 (1.67–3.93)	<0.001	—	—	—	—	—	—
History of peripheral vascular disease	—	—	—	—	2.62 (1.20–5.73)	0.015	—	—
History of hypertension	1.46 (1.02–2.10)	0.039	—	—	—	—	—	—
History of stroke or transient ischaemic attack	—	—	—	—	—	—	—	—
History of chronic obstructive pulmonary disease	—	—	1.36 (0.96–1.93)	0.082	—	—	—	—
History of diabetes mellitus	—	—	1.31 (0.97–1.77)	0.078	—	—	1.46 (1.02–2.07)	0.037
Preoperative estimated glomerular filtration rate <60 ml min^−1^ (1.73 m^2^)^−1^	3.68 (2.29–5.91)	<0.001	1.67 (1.10–2.53)	0.016	1.90 (1.14–3.15)	0.013	1.67 (1.05–2.66)	0.029
High-risk surgery	—	—	—	—	1.44 (1.05–1.98)	0.025	—	—
CI <0.6	1.57 (1.11–2.23)	0.011	1.40 (1.09–1.81)	0.009	7.57 (5.50–10.43)	<0.001	2.63 (1.97–3.52)	<0.001

### Primary outcome: myocardial injury

Within 3 days after surgery, 162/1325 (12.2%) patients sustained myocardial injury, which occurred in 50/396 (12.6%) patients with CI <0.6 and 112/928 (12.1%) patients with CI ≥0.6. There was no difference in the odds of myocardial injury amongst patients with CI <0.6 compared with those with CI >0.6 (unadjusted OR: 1.05 [0.74–1.50]; *P*=0.78). In the multivariable analysis, CI <0.6 was not associated with myocardial injury (*P*>0.60).

### Secondary outcome: sympathetic *vs* parasympathetic measures and myocardial injury

We examined the prevalence of physiological markers of impaired sympathetic and parasympathetic functions using chronotropic incompetence and HR recovery, respectively. We found that 169 (12.8%) had low CI alone, 294 (22.2%) had HR recovery <12 beats min^−1^ alone, and 227 (17.2%) had both HR recovery <12 beats min^−1^ and CI <0.6. When we repeated the primary analysis using HR recovery <12 beats min^−1^ and CI <0.6 as the exposures, we found that only HR recovery <12 beats min^−1^ was associated with myocardial injury (Supplementary Table S1).

### Secondary outcome: postoperative mortality

**Fig 2:**
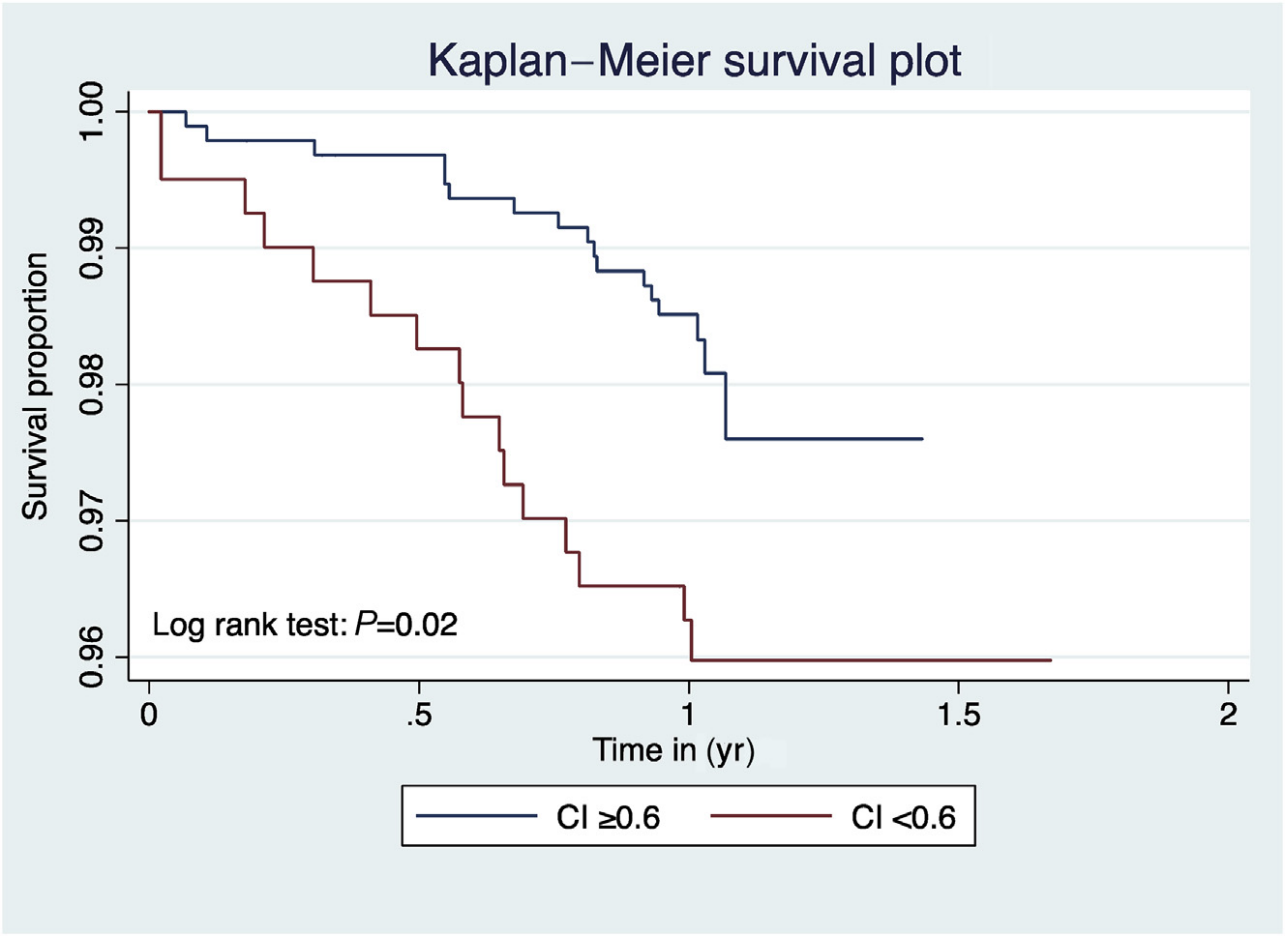
Kaplan–Meier survival plot for chronotropic incompetence (chronotropic index <0.6) *vs* no chronotropic incompetence (chronotropic index ≥0.6).

Within 1 yr of surgery, 33/1325 (2.5%) patients had died. With univariable analysis, postoperative mortality was more frequent amongst patients with CI <0.6 (16/396 [4.0%]) compared with patients without CI <0.6 (17/928 [1.8%]; unadjusted OR: 2.26 [1.13–4.51]; *P*=0.02. However, on multivariable analysis, CI <0.6 and mortality were not significantly associated (OR: 1.98 [0.97–4.02]; *P*=0.06; Table 2 and Fig. 2).

### Sensitivity analyses

When we repeated the primary and secondary analyses, including the preoperative use of beta blockers, diltiazem, or verapamil as co-variates, the results were very similar (Supplementary Table S2). Similar proportions of patients receiving these drugs achieved RER (carbon dioxide produced/oxygen consumed) >1.05. When we repeated the primary analysis excluding patients receiving beta blockers, diltiazem, or verapamil, CI <0.6 was not associated with myocardial injury (OR: 7.20 [0.60–87.02]; *P*=0.12) in the univariable analysis. The multivariable model did not converge because of collinearity between variables. We could not complete the regression analysis for mortality because an insufficient number of patients died. When we repeated the primary analysis using age-predicted maximum HR calculated using the method described by Tanaka and colleagues,^23^ CI <0.6 was not associated with myocardial injury (OR: 1.10 [0.78–1.53]; *P*=0.59) or mortality (OR: 1.50 [0.75–2.99]; *P*=0.25) in the univariable analysis. In multivariable analysis, CI <0.6 was removed from the stepwise models at the *P*>0.56 level for both outcomes. When we repeated the analysis using CI <0.8 as the exposure, CI <0.8 was not associated with myocardial injury (OR: 0.87 [0.62–1.21]; *P*=0.41) or mortality (OR: 1.15 [0.56–2.36]; *P*=0.70) in the univariable analysis. In the multivariable analysis, CI <0.8 was removed from the stepwise model at the *P*>0.19 level (myocardial injury) and the *P*>0.97 level (mortality). When we repeated the analysis using CI as a continuous variable, chronotropic incompetence was not significantly associated with myocardial injury (OR: 1.20 [0.68–2.09]; *P*=0.53) or mortality (OR: 0.78 [0.38–1.62]; *P*=0.51).

## Discussion

The principal finding of this analysis was that preoperative chronotropic incompetence—an impaired ability to increase HR in response to exercise—was not associated with myocardial injury within 3 days after surgery. Impaired HR recovery (indicative of parasympathetic dysfunction) after exercise, rather than impaired HR increase during exercise (indicative of sympathetic dysfunction), was associated with postoperative myocardial injury. We also confirmed the deconditioned phenotype of subclinical cardiac failure in preoperative patients, as chronotropic incompetence was associated with elevated preoperative NT pro-BNP, a preoperative risk factor for postoperative cardiovascular morbidity and a biomarker for heart failure in the general population. Moreover, we found a strong association between chronotropic incompetence and CPET-derived markers for heart failure.^8^, ^34^ These data confirm our previous findings in a large prospective cohort that almost one-third of patients undergoing noncardiac surgery exhibit a phenotype of subclinical cardiac failure that is frequently accompanied by significant autonomic impairment.^8^, ^31^

We defined chronotropic incompetence using an established threshold of CI, which is prognostically associated with increased mortality in longitudinal cohorts of patients with heart failure.^21^ Our results do not support a link between beta-adrenoceptor dysfunction, as identified using chronotropic incompetence, and myocardial injury. The inability to increase HR in patients with chronotropic incompetence suggests that a direct link between HR and supply-demand mismatch is unlikely to underpin myocardial injury. However, it is plausible that chronotropic incompetence could promote myocardial injury through indirect links. The failure to increase cardiac output under certain perioperative circumstances, which require HR elevation, may be linked to myocardial injury, as suggested by the Perioperative Ischemic Evaluation (POISE) trial of perioperative metoprolol.^35^ Similarly, failure to meet metabolic demands during surgery may drive organ injury, which in turn could increase the risk of myocardial injury. The precise mechanism leading to a decrease in β_1_-adrenergic receptor expression and desensitisation in cardiac failure is unclear, but may involve oxidative stress^36^ driven by chronic systemic inflammation.^37^ Our finding that chronotropic incompetence is associated with reduced survival after noncardiac surgery is consistent with similar observations in patients with heart failure,^12^, ^21^, ^22^, ^38^, ^39^, ^40^ supporting the hypothesis that there is a cohort of surgical patients with severe, yet subclinical, heart failure.^8^

A notable strength of our study is that the results have high external validity attributable to the prospective, international, multicentre nature of the study cohort, which makes our findings readily generalisable to the majority of intermediate- and high-risk surgical patients. The primary outcome, myocardial injury, is an objective, biomarker defined endpoint and not subject to observer bias. Clinicians at each participating hospital were blinded to the results of the preoperative CPET. Therefore, the measurement of chronotropic incompetence did not influence perioperative care.

Our analysis also has several limitations. As with any observational study, it is possible that our results may be influenced by unmeasured confounding. The primary outcome was myocardial injury, and the sample size for the study, which was based on cardiovascular outcomes, was appropriate for this outcome. However, the study was not powered to detect differences in mortality, and therefore, we advise that inferences regarding mortality should be with caution.

It is possible that our results could have been influenced by the definition of chronotropic incompetence. We defined this as CI <0.6, which is an indicator of poor prognosis in patients with heart failure.^8^ However, some studies in other populations have used a different threshold of CI (<0.8).^33^ When we repeated the analysis using CI <0.8, the results were similar. CI was calculated as the proportion of age-predicted maximum HR reached during preoperative exercise. As with any pragmatic study of exercise, there is an underlying assumption that the HR recorded at peak exertion is an accurate measure of maximal HR. Because of the clinical nature of the study, we were unable to confirm this with repeated measurements, so there is a possibility that some measurements of maximum HR might not represent true maximal values. However, more than 80% of the cohort achieved an end-exercise RER (carbon dioxide produced/oxygen consumed) of >1.05, which is generally accepted to represent peak effort.^12^

There are several methods for calculating age-predicted maximum HR, which could potentially influence the results. We chose the method described by Astrand,^24^ which is the most widely accepted, as the primary method. However, we recalculated age-predicted maximum HR using the Tanaka and colleagues^23^ method, and the results were similar.^23^ When we repeated the analysis using CI as a continuous variable, we did not identify a relationship with myocardial injury. However, this method assumes a linear relationship between CI and the risk myocardial injury, which may not be true.

Resting HR or change in HR may be influenced by rate-limiting medications. When we repeated the analysis after removing the 224 patients receiving beta blockers or rate-limiting calcium channel antagonists, our results were similar. We also repeated the multivariable analysis including treatment with beta blockers or rate-limiting calcium channel anatagonists as separate terms in the model, and our results were similar. CPET were conducted and interpreted by investigators at 24 participating hospitals, so there is a potential for observer bias and measurement error between centres. However, this was mitigated through the prospective use of a standardised CPET protocol and case report form.^16^ It is possible that a potential relationship between chronotropic incompetence and myocardial injury may have been confounded by intraoperative hypotension. However, when we repeated the primary analysis adding intraoperative vasopressor use (a surrogate marker of hypotension) as a co-variate, the results were similar.

### Conclusions

Chronotropic incompetence was associated with both impaired cardiopulmonary function and elevated NT pro-BNP (indicating subclinical heart failure). However, in contrast to parasympathetic measures, chronotropic incompetence was not linked to myocardial injury. These data suggest that a mechanistic role for sympathetic dysregulation in myocardial injury is unlikely, and adds further support to the hypothesis that cardiac vagal dysfunction is the predominant autonomic influence in determining myocardial injury and perioperative outcome.^5^, ^8^, ^31^, ^32^, ^41^, ^42^

## Authors' contributions

Hypothesis conception: TEFA, GLA

Analysis plan design: TEFA, RMP, BHC, DW, GLA

Data analysis: TEFA, GLA

Writing paper: TEFA, GLA with input from RMP

Revising paper: all authors

## Declaration of interest

The Measurement of Exercise Tolerance before Surgery study funding sources had no role in the design and conduct of the study; collection, management, analysis, and interpretation of the data; and preparation or approval of the article. RMP holds research grants, and has given lectures and performed consultancy work for Nestlé Health Sciences, B. Braun, Medtronic, GlaxoSmithKline, and Edwards Lifesciences, and is a member of the associate editorial board of the *British Journal of Anaesthesia*. GLA is a member of the editorial advisory board for *Intensive Care Medicine Experimental*, is an Editor for the *British Journal of Anaesthesia*, and has undertaken consultancy work for GlaxoSmithKline. TEFA is a committee member of the Perioperative Exercise Testing and Training Society. MPWG is Vice President of CPX International, serves on the medical advisory board for Sphere Medical Ltd, and is joint editor-in-chief of *Perioperative Medicine*. There are no other relationships or activities that could appear to have influenced the submitted work.

## Funding

Medical Research Council and *British Journal of Anaesthesia* clinical research training fellowship (MR/M017974/1 to TEFA); UK National Institute for Health Research Professorship (to RMP); *British Journal of Anaesthesia* / Royal College of Anaesthetists basic science career development award, British Oxygen Company research chair grant in anaesthesia from the Royal College of Anaesthetists, and British Heart Foundation Programme Grant (RG/14/4/30736 to GLA); merit awards from the Department of Anesthesia at the University of Toronto (to BHC and DW); New Investigator Award from the Canadian Institutes of Health Research (to DW) ; --> NIHR Southampton Biomedical Research Centre (to MPWG)
