# Supplementary material for: Chronotropic incompetence and myocardial injury after noncardiac surgery: planned secondary analysis of a prospective observational international cohort study
Source: Br J Anaesth. 2019 Apr 24;123(1):17–26. doi: 10.1016/j.bja.2019.03.022 (PMC6676775; doi:10.1016/j.bja.2019.03.022)
Supplement: Multimedia component 1 [file mmc1.docx]

**Chronotropic incompetence and myocardial injury after non-cardiac surgery: results of a prospective international cohort study**

**SUPPLEMENTARY FILE**

**T. E. F. Abbott,^1^ R. M. Pearse,^1^ W. S. Beattie,^2^ M. Phull,^3^ C. Beilstein,^4^ A. Raj,^5^ M. P. W. Grocott,^6^ B. H. Cuthbertson,^2, 7^ D. Wijesundera^2, 8^ and G. L. Ackland.^1^**

1. *William Harvey Research Institute, Queen Mary University of London, UK*
2. *Department of Anesthesia, University of Toronto, Toronto, Ontario, Canada.*
3. *Department of Intensive Care Medicine, Queens Hospital, Romford, UK*
4. *Department of Anaesthesiology and Pain Therapy, Bern University Hospital, 3010 Bern, Switzerland*
5. *Department of Intensive Care Medicine, Croydon University Hospital, Croydon, UK*
6. *Critical Care Research Group, Southampton NIHR Biomedical Research Centre, University Hospital Southampton, University of Southampton, Southampton, UK*
7. *Department of Critical Care Medicine, Sunnybrook Health Sciences Centre, 2075 Bayview Avenue, Toronto, Ontario, Canada*
8. *Li Ka Shing Knowledge Institute, St. Michael's Hospital, Toronto, Ontario, Canada.*

Correspondence to:

Gareth L. Ackland PhD FRCA FFICM

Translational Medicine and Therapeutics

William Harvey Research Institute

Queen Mary University of London

London EC1M 6BQ

e-mail: g.ackland@qmul.ac.uk

Tel: +44 207 882 2107

**Keywords: Observational study; heart rate; Surgery**

**Supplementary table 1. Chronotropic Incompetence and impaired heart rate recovery.**

**The cohort was stratified by chronotropic incompetence <0.6 (CI<0.6) and impaired heart rate recovery < 12 beats per minute (HRR<12), to give four groups. The independent variable was this four-level categorical variable. The dependent variable was firstly myocardial injury and secondly mortality within the one-year follow-up period. The analysis was not adjusted for other factors. Results of two separate analyses are presented. The reference category was CI≥0.6 and HRR≥12. Results are presented as odds ratios with 95% confidence intervals and associated p-values.**

| **Covariates** | **odds ratio** | **p-value** |
| --- | --- | --- |
| **Myocardial injury** |  |  |
| CI≥0.6 and HRR≥12 | - | - |
| CI<0.6 and HRR≥12 | 0.78 (0.43-1.42) | 0.41 |
| CI≥0.6 and HRR<12 | 1.60 (1.06-2.39) | 0.02 |
| CI<0.6 and HRR<12 | 1.62 (1.05-2.51) | 0.03 |
|  |  |  |
| **Mortality** |  |  |
| CI≥0.6 and HRR≥12 | - | - |
| CI<0.6 and HRR≥12 | 2.58 (1.03-6.41) | 0.04 |
| CI≥0.6 and HRR<12 | 0.90 (0.31-2.57) | 0.84 |
| CI<0.6 and HRR<12 | 1.89 (0.76-4.69) | 0.17 |
|  |  |  |

**Supplementary table 2. Sensitivity analysis including the use of preoperative beta-blockers, or heart rate-limiting calcium channel antagonists as covariates.**

**Results of two separate analyses are presented. Results are presented as odds ratios with 95% confidence intervals and associated p-values. CI<0.6 was rejected from the multivariable analysis for myocardial injury (p>0.55) so the results of the univariable analysis are presented. Transient ischaemic attack (TIA); Chronic Obstructive Pulmonary Disease (COPD).**

| **Analysis** | **odds ratio** | **p-value** |
| --- | --- | --- |
| **Myocardial injury (univariable)** |  |  |
| CI<0.6 | 1.05 (0.74-1.50) | 0.78 |
|  |  |  |
| **Mortality (multivariable)** |  |  |
| Stroke or TIA | 2.99 (1.00-9.02) | 0.05 |
| COPD | 2.61 (1.17-5.86) | 0.02 |
| CI<0.6 | 1.98 (0.97-4.01) | 0.06 |
| Male sex | 2.22 (0.98-5.03) | 0.06 |
|  |  |  |

**Sup. Figure 1. Mortality within the one-year follow-up period stratified by tertiles of chronotropic index. Error bars show 95% confidence intervals.**

**
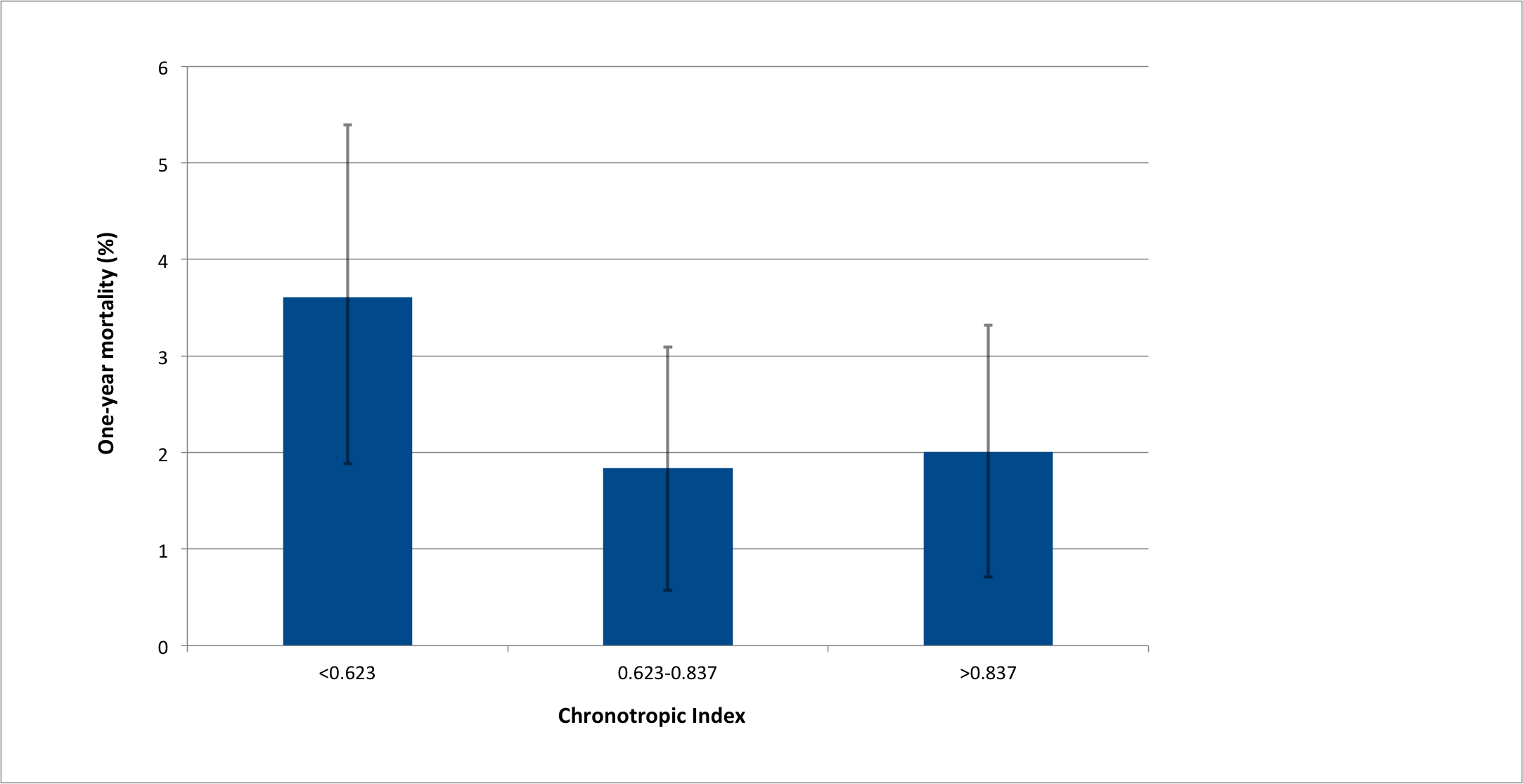
**
